# Are patient education and self‐care advantageous for patients with head and neck cancer? A feasibility study

**DOI:** 10.1002/nop2.361

**Published:** 2019-08-24

**Authors:** Anne Söderlund Schaller, Elena Dragioti, Gunilla M. Liedberg, Britt Larsson

**Affiliations:** ^1^ Pain and Rehabilitation Centre and Department of Medical and Health Sciences Linköping University Linköping Sweden; ^2^ Division of Occupational Therapy, Department of Social and Welfare Studies Faculty of Health Sciences, Campus Norrkoping, Linköping University Linköping Sweden

**Keywords:** education, head and neck cancer, pain, psychological symptoms, quality of life, self‐care

## Abstract

**Aim:**

This study evaluates whether patient education and individually self‐care reduces pain and improves QoL, mood and sleep during and after radiotherapy treatment for patients with head and neck cancer.

**Design:**

A longitudinal, two‐armed feasibility study design was performed.

**Methods:**

Sixty‐four participants with curative intent were included in the study. All participants answered questions about pain three times a week and completed a survey questionnaire about pain, QoL, psychological aspects and barriers towards pain management at baseline, at 4 weeks and at 10 weeks. Thirty‐four of the participants attended in two education sessions on pain based on their beliefs about pain and received individualized self‐care instructions based on their weekly rating of pain.

**Result:**

This study did not find any significant group differences for the pain, QoL, mood and sleep.

## 1 INTRODUCTION

### 1.1 Background and objectives

Pain prevalence in patients with head and neck cancer (HNC) is 50%–80% and related to tumour, surgery, chemotherapy and radiotherapy treatment (RT) (van der Molen et al., 2009). Poor pain management can be due to inadequate pain assessment and lack of knowledge among caregivers as well as among patients with cancer (Oldenmenger, Sillevis Smitt, van Dooren, Stoter, & van der Rijt, 2009). For example, despite severe pain, patients with cancer only used about half their prescribed medication (Miaskowski et al., 2001) and patients' beliefs such as fear of addiction, misunderstanding about dosages and feelings that it is not possible to treat the pain adequately can provide barriers to optimal management of cancer pain (Gunnarsdottir, Donovan, Serlin, Voge, & Ward, 2002; Ward et al., 1993). Systematic reviews conclude a decrease in pain intensity for patients with cancer is associated with education about pain (Bennett, Bagnall, & Jose Closs, 2009; Howell, Harth, Brown, Bennett, & Boyko, 2017; Jho, Myung, Chang, Kim, & Ko, 2013; Koller, Miaskowski, De Geest, Opitz, & Spichiger, 2012; Ling, Lui, & So, 2012; Marie, Luckett, Davidson, Lovell, & Lal, 2013). One systematic review concludes positive effects of education for patients with cancer on depression, anxiety and quality of life (QoL) (Howell et al., 2017). However, one review found no effect of patient education on QoL in patients with cancer (Ling et al., 2012). Educational interventions for sleep disturbance are sparse (Langford, Lee, & Miaskowski, 2012). Self‐care (SC) refers to what patients do on their own to achieve, maintain and promote optimal health (Denyes, Orem, Bekel, & SozWiss, 2001) and may decrease pain in several pain conditions (Du et al., 2011; Oliveira et al., 2012). Pain in patients with HNC has been reported to be difficult to treat with analgesics (Epstein et al., 2010; Ling & Larsson, 2011). Patients with HNC often suffer from symptoms that negatively affect QoL (Rogers et al., 2016), mood (Haisfield‐Wolfe et al., 2012) and sleep (Shuman et al., 2010).

The effect of education and SC on pain and other common symptoms in patients with HNC needs to be elucidated. This study evaluated whether patient education and individually tailored SC reduces pain intensity and improves QoL, mood and sleep during and after RT treatment.

We hypothesized that individually tailored patient education and adapted SC can help reduce pain, maintain QoL, stabilize mood and improve sleep in patients with HNC during and after RT.

## 2 METHODS

### 2.1 Trial design

This two‐armed feasibility study compared patient education on management of cancer pain in combination with advice on SC. This trial is registered in ClinicalTrials.gov Identification NCT03089736.

### 2.2 Participants

The participants were patients with HNC undergoing RT and referred to the Pain and Rehabilitation Centre (PRC) (University Hospital, Linköping, Sweden) for anticipated pain. It was not possible to include patients in the present study before the start of RT. The Swedish law restricts contact to patients before enrolment to the PRC. The patients were included within 2 weeks after receiving ongoing RT. The following inclusion criteria were used as follows: able to read, write and understand Swedish, registered in RT with curative intent and 18 years of age or older. In connection with scheduled RT, verbal and written information about the study was provided to all available patients by trained research nurses (TRN). After 1 week, the eligible patients were asked whether they wanted to be included in the study. Data in this study were collected as short message services (SMS) and at the PRC.

### 2.3 Interventions

#### 2.3.1 Both groups

All participants answered a SMS with seven items on pain intensity and interference every Monday, Wednesday and Friday during the 10‐study weeks. If the SMS survey showed ≥3 numeric rating scales step increase on any items, a TRN phoned the patient the same day (no later than 3 days if a weekend). Based on the SMS scores, pharmacological treatment was promptly prescribed or adjusted. If the patient was displeased with the pain relief at the next day's phone contact with a TRN (or at next scheduled individual weekly follow‐up if imminent), the pharmacological treatment was adjusted. Both groups were offered care as usual at the PRC. Thus, they were encouraged to contact the TRNs by phone and had access to advice from the TRNs. The pharmacological treatment was based on identical principles for both groups and prescribed by the physicians according to the ward's policies (Appendix S1).

#### 2.3.2 The intervention group

##### Two‐tailored Patient Education Sessions

The scientific literature (Koller et al., 2012; Lovell et al., 2014) on patient education on management of cancer pain was scrutinized. Six essential education areas were identified: pain and pain physiology, pain medication, side effects of medication and prevention of side effects, abuse of medications and advice on sleep and anxiety. To make it possible to tailor education interventions for each patient, a procedure to match The Barriers Questionnaire II (BQ‐II) items to the six educational areas was undertaken. Thus, the BQ‐II scoring of the items (several items could be assigned the same education area) coordinated by most of a group of nurses (10 experienced nurses employed at PRC and the first author [AS]) to each education area (Table 1) constituted the base for the individual tailoring of education. The inter‐rater reliability of the item‐to‐education coordination process was measured using a two‐way mixed, consistency, average‐measures intraclass correlation (ICC) to assess the degree that the 11 coders provided consistency in their ratings of education areas across the 27 items of the BQ‐II. The ICC was .91. A minimum summed BQ‐II score (Table 1) of the group of items assigned to each education area decided whether and which education should be delivered.

**Table 1:** Items of the Barriers Questionnaire II (BQ‐II) and corresponding education area

Items in BQ‐II (number of the item in BQ‐II)	Education area (least average score of adjacent items for the education to be offered)
Confusion from pain medicine cannot be controlled (5) Using pain medicine blocks your ability to know if you have any new pain (7) If you take pain medicine when you have some pain, then it might not work as well if the pain becomes worse (15) Pain medicine can keep you from knowing what's going on in your body (16) If you use pain medicine now, it won't work as well if you need it later (21) Pain medicine can mask changes in your health (22)	Pain and pain physiology (12)
When you use pain medicine, your body becomes used to its effects and soon it won't work anymore (6) Using pain medicine blocks your ability to know if you have any new pain (7) Pain medicine makes you say or do embarrassing things (14) If you take pain medicine when you have some pain, then it might not work as well if the pain becomes worse (15) Pain medicine can keep you from knowing what's going on in your body (16) If you use pain medicine now, it won't work as well if you need it later (21) Pain medicine can mask changes in your health (22)	Pain medication (14)
Drowsiness from pain medicine is difficult to control (3) Nausea from pain medicine cannot be relieved (10) Using pain medicine can harm your immune system (13) Pain medicine makes you say or do embarrassing things (14) Constipation from pain medicine cannot be relieved (17) Pain medicine can hurt your immune system (19) It is easier to put up with pain than with the side effects that come from pain medicine (20)	Side effects and prevention of side effects (14)
There is a danger of becoming addicted to pain medicine (2) Many people with cancer get addicted to pain medicine (9) Pain medicine is very addictive (23)	Abuse about medications (6)
Cancer pain can be relieved (1) Pain medicine can effectively control cancer pain (8) Medicine can relieve cancer pain (24)	Advice on anxiety (reverse score: items 1, 8, 24 [<9])
Pain medicine weakens the immune system (4) It is important to be strong by not talking about pain (11) It is important for the doctor to focus on curing illness and not waste time controlling pain (12) If doctors have to deal with pain, they won't concentrate on curing the disease (18) Pain medicine can mask changes in your health (22) Doctors might find it annoying to be told about pain (25) Reports of pain could distract a doctor from curing the cancer (26) If I talk about pain, people will think I'm a complainer (27)	Advice on anxiety (16)
How satisfied are you with your current sleep (28)	Advice on sleep (2)

At week 1 (baseline [BL]), a TRN delivered a PowerPoint presentation covering the education areas. This presentation was labelled education session 1 (ES 1). To ensure that as many current needs as possible were addressed at week 4 (ES 2), the TRN initiated a structural discussion with the patient on the specific education areas presented at ES1. If needed, based on the second scoring of BQ‐II 1 week before the ES 2, additional education areas were presented at ES 2.

##### Individually tailored self‐care

The scientific literature of SC for patients with cancer was reviewed (Johnston et al., 2009; Wong et al., 2006; Worthington et al., 2011), which was supplemented with the first authors and six nurses employed at PRC clinical experiences of pain care regarding patients with HNC. Fourteen SC measures were identified that covered maintaining well‐being, prevention of symptoms and pain relief of mouth and throat (Appendix S2). At weekly follow‐ups at the PRC, adjustments or initiations of SC were suggested depending on the previous three SMS scores. As the intention was a preventive approach, SC measurement was systematically selected and suggested (Table 2) if the score on any of the SMS items was ≥3 (except ≥1 on the item pain interferences on general activities). In addition, the TRN verbally presented and provided structured and detailed written information on the recommended SC (Appendix S2).

**Table 2:** Self‐care measurements recommended at weekly follow‐up when NRS score ≥3 on any item (≥1 on the item pain interferences on general activities) of the short message services (SMS) scores

Items on pain three times weekly by SMS	Self‐care instruction recommended^a^
Pain intensity	
When talking, eating and drinking	3–4, 9–11
When not talking, eating or drinking	3–4, 9–11
Pain interference	
General activities	1–4
Mood	1–2, 8
Relations with other people	8
Sleep	5–7
Enjoyment of life	1–2, 8

a Number of self‐care refers to numbers in Appendix S2.

#### 2.3.3 The control group

The control group did not receive the individual tailored education sessions and the systematic adjustments of SC at weekly follow‐up. However, the weekly follow‐up of the control group was consistent with the usual care at the PRC. That is, if the patient asked for advice or if it was apparent to a TRN that one or more unstructured SCs would be beneficial, verbal advice regarding SC that the TRN came to think of was provided. This was necessary for ethical reasons.

### 2.4 Outcomes

#### 2.4.1 Primary outcomes

##### Pain intensity and pain interference

The primary outcome measurements included seven items about pain intensity and pain interference the previous 24 hr reported by both groups of patients and collected by SMS every Monday, Wednesday and Friday during the 10‐study weeks.

The University of California San Francisco (UCSF) Oral Cancer Pain Questionnaire (Connelly & Schmidt, 2004) measures pain experiences from the oral cavity and consists of eight items scored on a scale from 0 (no pain)–10 (the most intense pain). In the SMS survey, two items from the Oral Cancer Pain Questionnaire were added on pain intensity in connection with and without speaking, talking and drinking. The Oral Cancer Pain Questionnaire is valid for patients with oral cancer pain (Connelly & Schmidt, 2004; Kolokythas, Connelly, & Schmidt, 2007).

The Brief Pain Inventory (BPI) measures two targets: the subjective intensity of pain and how pain interferes with daily activities (Cleeland & Ryan, 1994). The BPI consists of 12 items: five items related to pain intensity and seven items related to pain interference on function both rated on a 0 (no interference)–10 (interferes completely) scale. In the SMS survey, five items on pain interference from the BPI were included general activities, mood, relations, sleep and enjoyment of life. The BPI instrument has been validated for patients with cancer (Cleeland & Ryan, 1994; Kumar, 2011).

For each subscale and item, the average score of the three weekly scores was calculated.

#### 2.4.2 Secondary outcomes

A survey questionnaire including seven validated patient‐reported outcome measurements was used to collect the outcomes. Answered at BL, at 4 weeks and at 10 weeks, the secondary outcomes cover QoL, pain intensity, pain interference, psychological aspects and barriers towards pain management. A part of the secondary outcomes BL data are reported elsewhere (Schaller, Dragioti, Liedberg, & Larsson, 2017).

##### Quality of life

The Euro QoL‐5 Dimension Questionnaire (EQ‐5D) assesses health outcome and perceived state of health (Brooks, 1996). The questionnaire comprises five items: mobility, self‐care, usual activities, pain and discomfort, anxiety and depression. Each item has three response scales – no problems, some problems and extreme problems – and the answers were coded 1–3. An algorithm developed for EQ‐5 D was used to calculate the final individual score. The EQ‐5D score has a range from −0.5–1, where negative values mean low QoL and 1 means no reduction in QoL. The EQ‐5D scores were determined by applying scores from standard population values (Dolan, 1997). The second part of the EQ‐5D is the Euro Quality of Life Vertical Visual Analogue Scale (EQ‐VAS), which measures the respondent's general health on a vertical visual analogue line with 100‐scale steps with the endpoints labelled “Best imaginable health state” and “Worst imaginable health state” (Fayers & Machin, 2013).

The EQ‐5D, a valid and reliable instrument (Coons, Rao, Keininger, & Hays, 2000), was selected because it is a generic instrument that can be used for patients with different conditions and diseases (Fayers & Machin, 2013).

##### Pain intensity and pain interference

The Brief Pain Inventory (BPI) measures intensity of pain and pain interference (see description above) (Cleeland & Ryan, 1994). The scores were summed, and mean values of the items of pain interference and pain intensity items were calculated. The Swedish version of BPI used in this study has been linguistically validated (Anderson, 2019) but has not yet been psychometrically validated.

##### Anxiety and depression

The Hospital Anxiety and Depression Scale (HADS) assesses anxiety and depression (Bjelland, Dahl, Haug, & Neckelmann, 2002). This scale consists of 14 items: seven items are related to anxiety and seven to depression and is rated on a four‐point scale ranging from 0–3. The scores were summed, and the range for each subscale is 0–21. Higher scores indicate likelihood of anxiety or depressive symptoms. A score of 7 or less indicates a non‐case, a score of 8–10 a doubtful case and a score of 11 or more a definite case. HADS, a valid and reliable instrument (Bjelland et al., 2002; Zigmond & Snaith, 1983), is widely used in clinical practice, pain care and research and detects anxiety and depressive symptoms in a general setting.

##### Pain catastrophizing

The Pain Catastrophizing Scale (PCS) measures thoughts or feelings of catastrophizing in relation to how individuals experience pain (Sullivan, Bishop, & Pivik, 1995). The questionnaire comprises 13 items, including subscales for rumination, magnification and helplessness. Each item is scored on a five‐point scale from 0 (not at all)–4 (all the time). In this study, the total score was used and summed, and mean values of the items were calculated. The score range is 0–52, with higher scores indicating a worse situation. The PCS, a valid and reliable instrument (Osman et al., 2000), is used in clinical settings and research.

##### Barriers towards pain

The Barriers Questionnaire II (BQ‐II) comprises 27 items on patient‐reported beliefs on pain and pain management (Gunnarsdottir et al., 2002; Ward et al., 1993). Each item is measured on a six‐point scale – 0 (do not agree at all)–5 (agree very much) – with a total score of 0–135 with higher scores indicating higher barriers. Before this study, BQ‐II was translated into Swedish using a backward–forward procedure (Appendix S1, text 1). Cronbach's alpha coefficient was .90. The BQ‐II has been found to have good validity for patient‐related barriers to pain management in several studies involving patients with cancer (Gunnarsdottir et al., 2002; Valeberg et al., 2009; Ward et al., 1993).

##### Current sleep pattern

The Insomnia Severity Index (ISI), a self‐reported questionnaire, measures insomnia and provides a measure of the severity of sleep disorders (Bastien, Vallieres, & Morin, 2001). The ISI comprises seven items, and each item is rated on a scale from 0–4, and the total score ranges from 0–28. A higher score suggests more severe insomnia. In this study, one issue about sleep from the ISI was added at the end of the BQ‐II and reads as follows: How satisfied are you with your current sleep? The ISI has been found to be a valid and reliable instrument (Savard, Savard, Simard, & Ivers, 2005) and was used because sleep disorders are common in patients with pain.

### 2.5 Sample size and randomization

The sample size was assessed based on pain intensity (0–10 scale). With an assumed clinically relevant average difference in four scale steps (*SD* 3), an alpha value of .05 and a power of 80%, each group was calculated to include approximately 30 patients.

Every second patient of eligible patients was assigned to the control group (30 patients), and every second patient was assigned to the intervention group (34 patients).

### 2.6 Allocation concealment mechanism

When patients agreed to participate in the study, their personal data were documented in consecutive order on a list in a confidential data file. All patients were assigned a code number and were consecutively distributed to the intervention group or to the control group – every other patient to the control group and every other to the intervention group.

The first author generated the distribution sequence, registered participants and assigned participants to the intervention or control group.

### 2.7 Statistical methods

All the data were analysed using SPSS 23.0 for Windows (IBM Corp.). All tests were two‐tailed, and statistical significance was defined as a value of *p* ≤ .05. The data are presented as median or mean values with standard deviation (*SD*) or minimum and maximum values and as percentages (%). The differences between groups at baseline were tested by the Student *t* tests for continuous variables and chi‐square test for dichotomous variables. For the primary outcomes, first the average score was calculated for three time points every week to measure the primary outcomes for 10 weeks. Then, a linear‐repeated‐measures multilevel model (generalized estimating equations [GEE] continuous variables) was used to determine the effectiveness of the intervention compared with control conditions over time. The estimated impact (i.e. overall effect; Table 4) of treatment on the outcome in the GEE model reflects the “combined” within‐ and between‐subjects effects. The results are presented as regression coefficients (B) with 95% confidence intervals (CI) and can be interpreted as the time effect for the groups at a certain follow‐up compared with baseline. In multilevel analysis, missing scores do not need an imputation strategy, as this analysis is flexible in handling missing data. A repeated‐measures ANOVA/mixed model (continuous variables) with Bonferroni post hoc tests was used for the secondary outcomes.

## 3 RESULTS

**Figure 1:**
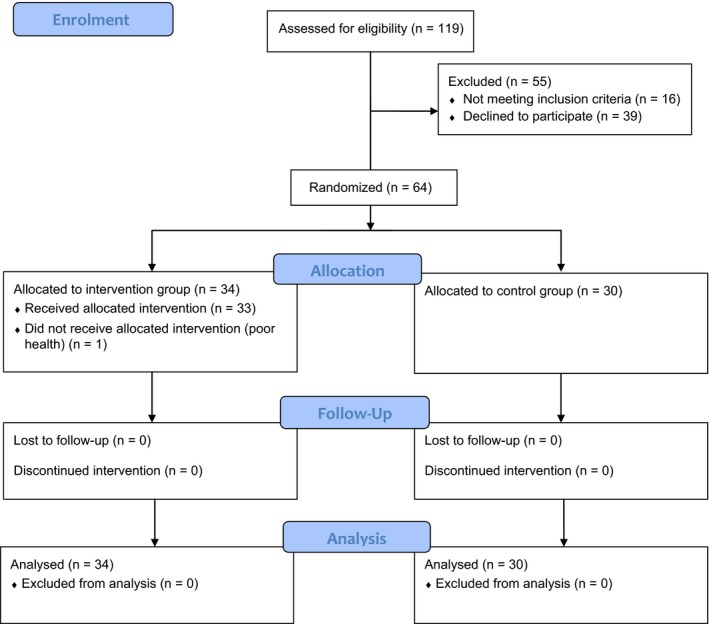
CONSORT flow chart of the recruitment process

A total of 119 patients were eligible, and 64 were selected (i.e., randomized) (Figure 1). The patients were randomly assigned to either the control group (30 patients) or the intervention group (34 patients). All 64 patients were analysed on an intention‐to‐treat basis. Excluded patients (*N* = 55) did not meet the inclusion criteria (*N* = 16) or declined to participate (*N* = 39). The process of inclusion was ongoing between January 2015–December 2016.

The 64 patients were diagnosed with HNC and informed on the curative intent of RT about 6.0 weeks (median) (min 2–max 711) before inclusion in the study.

The patients completed the first survey questionnaire in mean 7.4 days (*SD* 5.9 days) after the start of RT. Mean age of the participants was 65.1 years (*SD* 10.5 years), and the mean age of the non‐participants was 70.3 years (*SD* 12.8 years). Among the participants, 39 (60.9%) were men; the corresponding figure for the non‐participants was 31 (66.0%). The only reason reported for denying participating was poor health. Most participants cohabitated (42; 65.6%), most were former smokers (28; 43.8%), 10 (15.6%) were current smokers, and 26 (40.6%) had a university degree (Table 3). We previously have presented descriptive (and BL) data from most participants elsewhere (Schaller et al., 2017). Of the 34 patients, 33 (97.0%) in the intervention group completed the interventions. The reason for withdrawal was poor health.

**Table 3:** Participant characteristics at baseline

Characteristic; *N* (%), unless otherwise stated	Total (*N* = 64)	Intervention group (*N* = 34)	Control group (*N* = 30)	*p*‐value^a^
Age (years) (*M*, *SD*)	65.05 (±10.47)	64.0 (±10.42)	66.3 (±10.57)	.38
Women	25 (39.1)	12 (35.3)	13 (43.3)	.51
Living situation				
Not living alone	42 (65.6)	22 (64.7)	20 (66.7)	.86
Living alone	22 (34.4)	12 (35.3)	10 (33.3)	
Education				
Primary	10 (15.6)	4 (11.8)	6 (20.0)	.25
Second upper school/vocational	28 (43.8)	13 (38.2)	15 (50.0)	
College/University	26 (40.6)	17 (50.0)	9 (30.0)	
Smoking habits				
Non‐smokers	26 (40.6)	15 (44.1)	11 (36.7)	.83
Smokers	10 (15.6)	5 (14.7)	5 (16.7)	
Ex‐smokers	28 (43.8)	14 (41.2)	14 (46.7)	

Abbreviations: *M*, mean; *SD*, Standard deviation.

a Student *t* test for continuous variables or chi‐square test for categorical variables.

### 3.1 Primary outcomes

**Figure 2:**
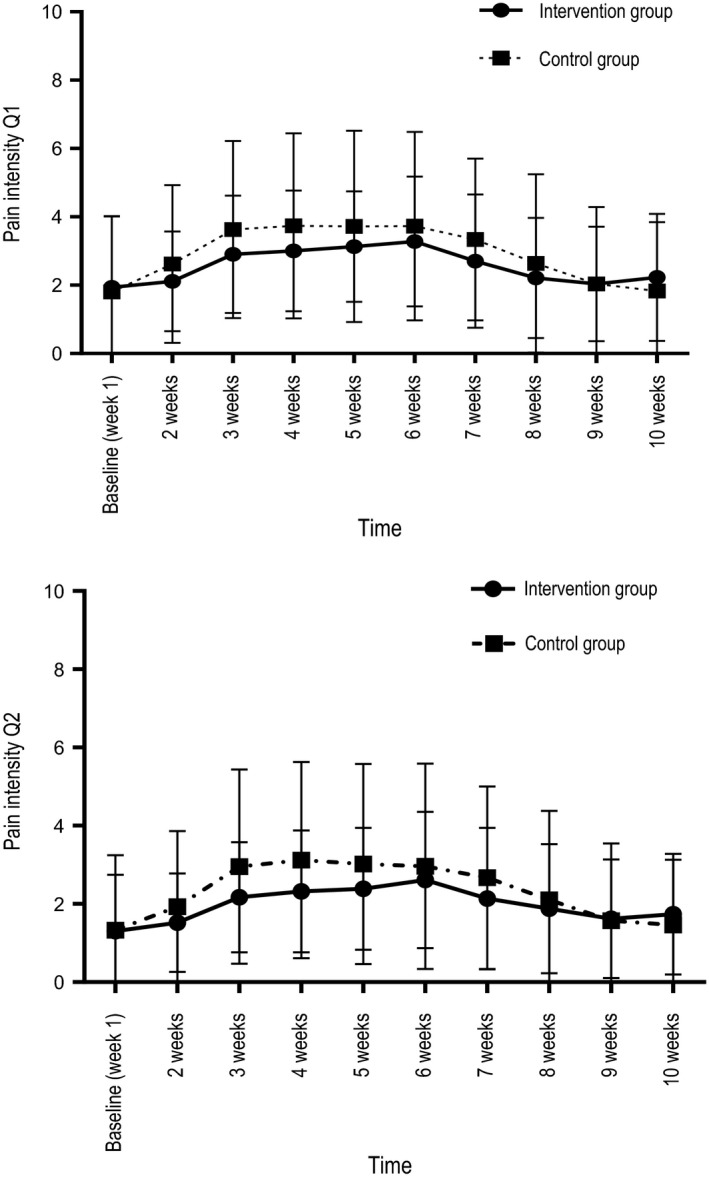
Scores of primary pain intensity outcomes by short message services (SMS) answers and standard deviation (vertical bars), for the intervention group (*N* = 34) and the control group (*N* = 30). Q1 = with speaking, talking and drinking, Q2 = without speaking, talking and drinking. Page 27, Figure 3, above the figure: Q1 = general activities, Q2 = mood, Q3 = relations, Q4 = sleep, Q5 = enjoyment of life

**Figure 3:**
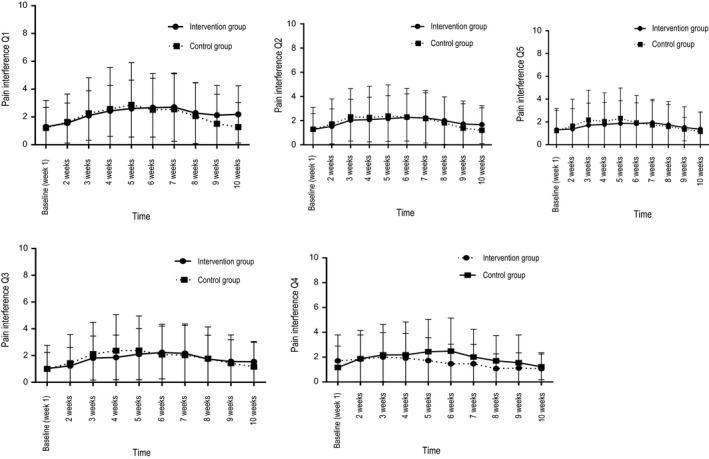
Scores of primary pain interference outcomes by short message services (SMS) answers and standard deviation (vertical bars), for the intervention group (*N* = 34) and the control group (*N* = 30)

We evaluated the effects of the intervention with education and SC on pain intensity and pain interference based on the SMS answers by performing repeated‐measures GEE model (Table 4; Figures 2 and 3). Missing values were 2%–3% from week 7 to week 10 with respect to all primary outcomes. The results showed no overall significant differences between the control and intervention groups over time.

**Table 4:** Mean scores (*SD*) and regression coefficients for primary outcomes by short message services answers

Variables	Intervention group (*N* = 34)	Control group (*N* = 30)	Regression coefficients (95% CI) B	*p*‐value
Pain intensity Q1 (0–10) (*M*, *SD*)				
Overall effect: *p* = 0.50				
1 week (baseline)	1.94 (2.09)	1.80 (2.22)	–	–
2 weeks	2.11 (1.46)	2.62 (2.31)	0.82 (0.36 to 1.28)	**.001**
3 weeks	2.90 (1.72)	3.63 (2.59)	1.83 (1.11 to 2.56)	**.000**
4 weeks	3.00 (1.77)	3.74 (2.71)	1.99 (1.13 to 2.84)	**.000**
5 weeks	3.13 (1.62)	3.72 (2.80)	1.94 (0.98 to 2.89)	**.000**
6 weeks	3.28 (1.90)	3.73 (2.76)	1.90 (0.95 to 2.84)	**.000**
7 weeks	2.70 (1.95)	3.34 (2.37)	1.38 (0.35 to 2.40)	**.009**
8 weeks	2.21 (1.76)	2.64 (2.61)	0.71 (−0.48 to 1.90)	.240
9 weeks	2.04 (1.68)	2.04 (2.26)	0.34 (−0.74 to 1.44)	.530
10 weeks	2.23 (1.86)	1.83 (2.03)	−0.19 (−1.32 to 0.95)	.743
Pain intensity Q2 (0–10) (*M*, *SD*)				
Overall effect: *p* = 0.36				
1 week (baseline)	1.30 (1.45)	1.33 (1.93)	–	–
2 weeks	1.52 (1.26)	1.93 (1.94)	0.60 (0.25 to 0.95)	**.001**
3 weeks	2.17 (1.41)	2.95 (2.49)	1.62 (1.06 to 2.19)	**.000**
4 weeks	2.32 (1.56)	3.12 (2.51)	1.87 (1.18 to 2.57)	**.000**
5 weeks	2.39 (1.56)	3.02 (2.56)	1.77 (1.02 to 2.52)	**.000**
6 weeks	2.61 (1.74)	2.96 (2.63)	1.71 (0.93 to 2.48)	**.000**
7 weeks	2.14 (1.81)	2.67 (2.33)	1.25 (0.36 to 2.14)	**.006**
8 weeks	1.88 (1.65)	2.10 (2.28)	0.84 (−0.13 to 1.80)	.090
9 weeks	1.62 (1.52)	1.57 (1.98)	0.44 (−0.46 to 1.35)	.338
10 weeks	1.74 (1.54)	1.46 (1.67)	−0.008 (−0.95 to 0.93)	.987
Pain interference Q1 (0–10) (*M*, *SD*)				
Overall effect: *p* = 0.83				
1 week (baseline)	1.29 (1.41)	1.21 (1.98)	–	–
2 weeks	1.56 (1.44)	1.64 (2.03)	0.43 (−0.19 to 1.05)	.176
3 weeks	2.10 (1.78)	2.26 (2.56)	1.05 (0.24 to 1.86)	**.011**
4 weeks	2.44 (1.83)	2.57 (2.99)	1.42 (0.43 to 2.41)	**.005**
5 weeks	2.60 (2.05)	2.87 (3.05)	1.73 (0.92 to 2.53)	**.000**
6 weeks	2.67 (2.12)	2.52 (2.61)	1.34 (0.50 to 2.18)	**.002**
7 weeks	2.70 (2.46)	2.55 (2.55)	1.26 (0.39 to 2.12)	**.004**
8 weeks	2.28 (2.19)	2.07 (2.38)	0.93 (−0.01 to 1.87)	.052
9 weeks	2.13 (2.14)	1.51 (2.13)	0.48 (−0.42 to 1.38)	.297
10 weeks	2.19 (2.06)	1.27 (1.78)	0.03 (−0.79 to 0.84)	.948
Pain interference Q2 (0–10) (*M*, *SD*)				
Overall effect: *p* = 0.73				
1 week (baseline)	1.28 (1.31)	1.29 (1.82)	–	–
2 weeks	1.53 (1.44)	1.72 (2.08)	0.43 (0.16 to 0.70)	**.002**
3 weeks	2.04 (1.73)	2.31 (2.36)	1.02 (0.56 to 1.48)	**.000**
4 weeks	2.09 (1.84)	2.26 (2.59)	1.09 (0.050 to 1.68)	**.000**
5 weeks	2.17 (1.89)	2.38 (2.59)	1.19 (0.46 to 1.91)	**.001**
6 weeks	2.26 (1.95)	2.30 (2.37)	1.11 (0.50 to 1.72)	**.000**
7 weeks	2.23 (2.08)	2.17 (2.34)	0.95 (0.34 to 1.56)	**.002**
8 weeks	2.00 (1.96)	1.82 (2.15)	0.68 (−0.01 to 1.38)	.054
9 weeks	1.72 (1.91)	1.38 (2.03)	0.43 (−0.32 to 1.18)	.259
10 weeks	1.67 (1.57)	1.20 (1.86)	0.29 (−0.36 to 0.94)	.386
Pain interference Q3 (0–10) (*M*, *SD*)				
Overall effect: *p* = 0.58				
1 week (baseline)	1.03 (1.21)	1.00 (1.76)	–	–
2 weeks	1.23 (1.39)	1.43 (2.14)	0.44 (0.90 to 0.80)	**.014**
3 weeks	1.81 (1.65)	2.11 (2.37)	1.12 (0.59 to 1.66)	**.000**
4 weeks	1.86 (1.67)	2.36 (2.70)	1.50 (0.75 to 2.24)	**.000**
5 weeks	2.10 (1.91)	2.38 (2.59)	1.50 (0.75 to 2.26)	**.000**
6 weeks	2.23 (1.98)	2.07 (2.27)	1.24 (0.59 to 1.88)	**.000**
7 weeks	2.16 (2.21)	2.03 (2.24)	1.18 (0.60 to 1.77)	**.000**
8 weeks	1.74 (1.79)	1.77 (2.37)	0.95 (0.21 to 1.69)	**.012**
9 weeks	1.55 (1.64)	1.42 (2.12)	0.87 (0.03 to 1.71)	.043
10 weeks	1.54 (1.52)	1.16 (1.83)	0.74 (−0.02 to 1.49)	.056
Pain interference Q4 (0–10) (*M*, *SD*)				
Overall effect: *p* = 0.25				
1 week (baseline)	1.70 (2.09)	1.17 (1.73)	–	–
2 weeks	1.84 (1.96)	1.88 (2.28)	0.71 (0.24 to 1.18)	**.003**
3 weeks	2.00 (1.97)	2.18 (2.47)	1.01 (0.47 to 1.55)	**.000**
4 weeks	1.92 (2.00)	2.19 (2.66)	1.07 (0.52 to 1.62)	**.000**
5 weeks	1.72 (1.86)	2.44 (2.62)	1.31 (0.68 to 1.94)	**.000**
6 weeks	1.46 (1.59)	2.49 (2.68)	1.44 (0.81 to 2.06)	**.000**
7 weeks	1.46 (1.56)	2.01 (2.24)	0.81 (0.36 to 1.26)	**.000**
8 weeks	1.08 (1.19)	1.70 (2.05)	0.81 (0.23 to 1.40)	**.007**
9 weeks	1.12 (1.23)	1.55 (2.24)	0.84 (0.12 to 1.55)	**.022**
10 weeks	1.07 (1.29)	1.22 (1.03)	0.69 (−0.08 to 1.46)	.078
Pain interference Q5 (0–10) (*M*, *SD*)				
Overall effect: *p* = 0.52				
1 week (baseline)	1.30 (1.72)	1.24 (1.96)		
2 weeks	1.39 (1.79)	1.64 (2.36)	0.40 (0.07 to 0.73)	**.019**
3 weeks	1.72 (1.93)	2.17 (2.62)	0.92 (0.46 to 1.39)	**.000**
4 weeks	1.78 (1.95)	2.04 (2.53)	0.95 (0.40 to 1.51	**.001**
5 weeks	1.87 (2.00)	2.31 (2.67)	1.20 (0.43 to 1.97)	**.002**
6 weeks	1.85 (1.83)	1.92 (2.41)	0.85 (0.27 to 1.44)	**.004**
7 weeks	1.93 (2.06)	1.73 (2.15)	0.60 (0.09 to 1.11)	**.022**
8 weeks	1.74 (1.81)	1.60 (2.19)	0.62 (−0.09 to 1.33)	.087
9 weeks	1.51 (1.83)	1.36 (1.03)	0.50 (−0.19 to 1.19)	.158
10 weeks	1.36 (1.51)	1.16 (1.72)	0.49 (−0.14 to 1.12)	.128

Note

Values presented are model estimates of generalized estimating equations models with a random intercept and adjusted for baseline. Regression coefficients can be interpreted as the time effect for the groups at a certain follow‐up moment compared with baseline. Significant differences are bold. The estimated impact (i.e. overall effect) of treatment reflects the “combined” within‐ and between‐subjects effects and Q = question. Pain intensity: Q1 = with speaking, talking and drinking, Q2 = without speaking, talking and drinking. Pain interference: Q1 = general activities, Q2 = mood, Q3 = relations, Q4 = sleep, Q5 = enjoyment of life.

Abbreviations: *M*, mean; *SD*, standard deviation.

As expected, time was associated with the primary outcomes (Table 4) in both groups. Compared with BL pain intensity, pain interference on mood and enjoyment of life were higher for weeks 2–7, pain interference on general activities was higher for weeks 3–7, and pain interference on relations with other people and on sleep was higher weeks 2–9. Student's *t* tests showed results identical to the GEE analyses.

### 3.2 Secondary outcomes

**Figure 4:**
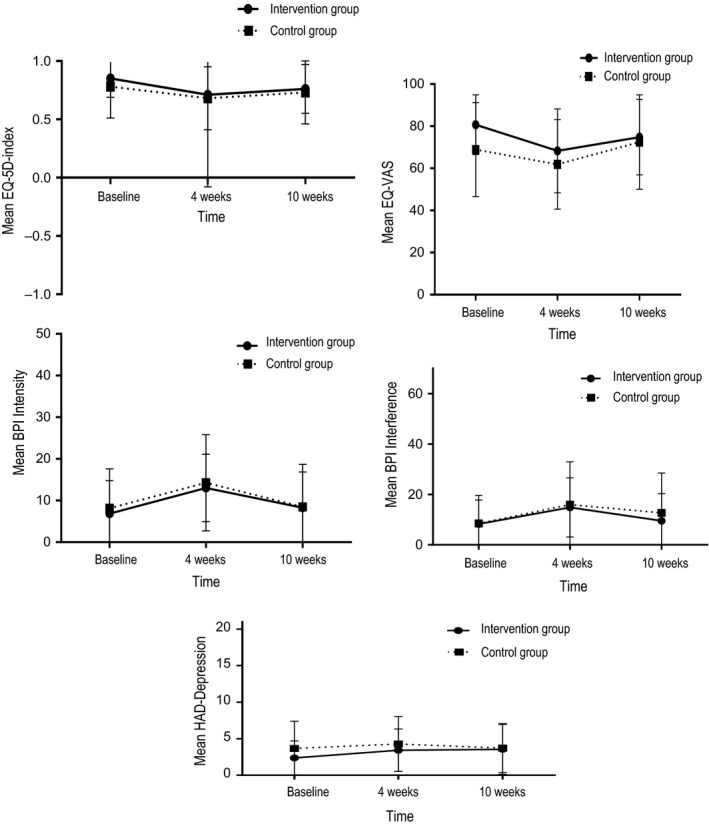
Scores of secondary outcomes and standard deviation (vertical bars), for the intervention group (*N* = 34) and the control group (*N* = 30)

**Figure 5:**
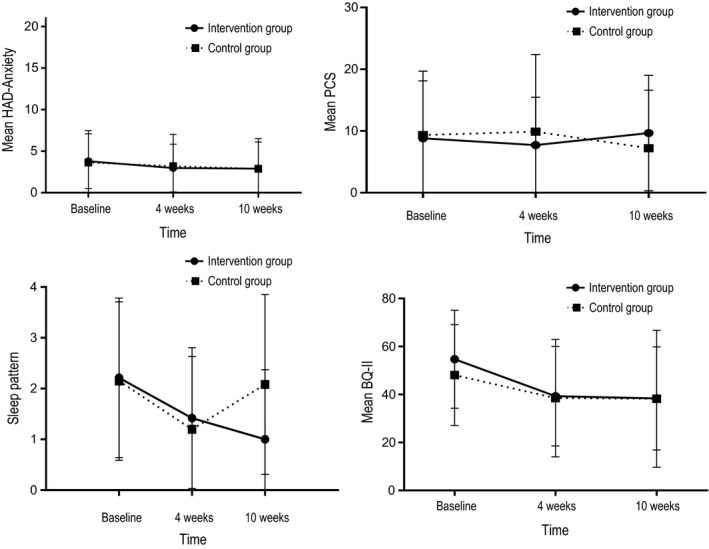
Scores of secondary outcomes with and standard deviation (vertical bars), for the intervention group (*N* = 34) and the control group (*N* = 30)

The effects of intervention with education and SC on the secondary outcomes were examined using mixed repeated‐measures ANOVA (Table 5; Figures 4 and 5). The between‐subjects factor consisted of the two groups (intervention and control), and the within‐subjects factor was three time points (BL, 4 and 10 weeks).

**Table 5:** Secondary outcomes results from baseline, 4 and 10 weeks and comparisons between‐ and within‐subjects effects (ANOVA)

Variables	Baseline mean (*SD*)		*p*‐value (between groups)	4 weeks, mean (*SD*)		*p*‐value (between groups)	10 weeks, mean (*SD*)		*p*‐value (between groups)	*p*‐value (within‐subjects effect over time)	Significant post hoc comparisons
	Intervention group (*N* = 34)	Control group (*N* = 30)		Intervention group (*N* = 34)	Control group (*N* = 30)		Intervention group (*N* = 34)	Control group (*N* = 30)			
EQ‐5D‐index (range −1–+1)	0.85 (0.16)	0.78 (0.27)	.20	0.71 (0.20)	0.68 (0.27)	.62	0.76 (0.21)	0.73 (0.27)	.71	**.003**	BL versus 4 weeks; ***p* = .003**
Overall effect: *p* = .57											
EQ‐VAS (range 0–100)	80.74 (14.24)	68.90 (22.34)	**.02**	68.28 (19.94)	61.88 (21.22)	.24	74.83 (17.92)	72.43 (22.41)	.67	**.000**	BL versus 4 weeks; ***p* = .000** BL versus 10 weeks; ***p* = .001**
Overall effect: *p* = .28											
BPI‐intensity (range 0–50)	6.85 (7.94)	8.13 (9.48)	.56	13.0 (8.12)	14.27 (11.56)	.64	8.28 (8.63)	8.46 (10.23)	.94	**.000**	4 weeks versus BL; ***p* = .000** 4 weeks versus 10 weeks; ***p* = .003**
Overall effect: *p* = .93											
BPI‐interference (range 0–70)	8.26 (9.57)	8.46 (11.18)	.94	14.82 (11.75)	15.96 (17.02)	.78	9.53 (10.85)	12.67 (15.83)	.41	**.001**	4 weeks versus BL; ***p* = .002** 4 weeks versus 10 weeks; ***p* = .037**
Overall effect: *p* = .91											
HAD‐anxiety (range 0–21)	3.79 (3.29)	3.63 (3.86)	.86	2.97 (2.88)	3.19 (3.85)	.80	2.90 (3.23)	2.88 (3.66)	.98	.454	NA
Overall effect: *p* = .59											
HAD depression (range 0–21)	2.38 (2.34)	3.67 (3.74)	.11	3.42 (2.90)	4.28 (3.76)	.35	3.55 (3.41)	3.72 (3.34)	.85	**.008**	BL versus 4 weeks; ***p* = .007**
Overall effect: *p* = .73											
PCS (range 0–52)	8.82 (9.33)	9.34 (10.38)	.83	7.70 (7.76)	9.88 (12.53)	.46	9.65 (9.37)	7.20 (9.42)	.34	.627	NA
Overall effect: *p* = .74											
Sleep pattern (range 0–4)	2.21 (1.57)	2.15 (1.56)	.88	1.42 (1.39)	1.20 (1.44)	.56	1.00 (1.37)	2.08 (1.77)	**.02**	**.002**	BL versus 4 weeks; ***p* = .001** BL versus 10 weeks; ***p* = .045**
Overall effect: *p* = .56											
BQ‐II (range 0–135)	54.67 (20.45)	48.10 (20.99)	.27	39.33 (20.78)	38.50 (24.47)	.90	38.38 (21.49)	38.26 (28.53)	.99	**.000**	BL versus 4 weeks; ***p* = .000** BL versus 10 weeks; ***p* = .001**
Overall effect: *p* = .11											

Notes

Significant differences are in bold. The estimated impact (i.e. overall effect) of treatment reflects the “combined” within‐ and between‐subjects effects.

Abbreviations: BL, baseline; BP I, Brief Pain Inventory; BQ‐II, Barrier Questionnaire; EQ‐5D, EuroQoL‐5‐Dimension Questionnaire; EQ‐VAS, Euro Quality of Life Vertical Visual Analogue Scale; HAD, Hospital Anxiety and Depression Scale; NA, not applicable; PCS, Pain Catastrophizing Scale; *SD*, standard deviation.

No statistically significant differences existed between the two groups except for higher EQ‐VAS at BL (Table 5) and sleep satisfaction at 10 weeks (Figure 4) in the intervention group.

For both groups, time was associated with the secondary outcomes (Table 5). Pain intensity and interference were significantly lower at BL compared with 4 weeks and decreased significantly between 4–10 weeks. Quality of life (EQ‐VAS) was statistically significantly higher at BL compared with 4 and 10 weeks. Depressive symptoms (HAD depression) were statistically significantly lower at BL compared with 4 weeks. Barriers to pain management (BQ‐II) were significantly lower at 4 and 10 weeks compared with BL. Student's *t* tests showed results identical to the mixed repeated‐measures ANOVA analyses.

## 4 DISCUSSION

This study did not find any significant group differences for the primary outcomes or for the secondary outcomes during RT. The only exception was sleep satisfaction, which was significantly higher in the intervention group at the end of RT. Although the QoL BL scores were significantly higher in the intervention group at BL, they were decreased to the similar level as the control group at the weeks 4 and 10. As expected for both groups, associations with time regarding all outcomes during the RT were found. To our knowledge, no study on pain education and SC for patients with HNC during RT has been published.

Our results are partly in line with previous RCTs on tailored pain education and SC during treatment including, for example, RT for patients with various cancer diseases that did not find any differences in pain intensity (Kravitz et al., 2011). The authors of a review on education and SC (Koller et al., 2012) conclude no improvements for outpatients, a finding that agrees with our study comprised entirely of outpatients. One review concluded, however, that patients with cancer reduced their pain after education and SC (Bennett et al., 2009). Consistent with previous research (Babin et al., 2008; Bennett et al., 2009; Ling et al., 2012), all patients in our study reported high QoL, which significantly decreased during RT. This was also the case for depressive symptoms. The literature is, however, contradictory regarding the effects of education and SC on depressive symptoms in patients with cancer (Dodd et al., 2010; Howell et al., 2017; Krischer, Xu, Meade, & Jacobsen, 2007).

The improvement on sleep satisfaction favouring the intervention group at week 10 should be treated with caution as the overall results do not point to significant effects of education and SC, and therefore, this could represent a random finding. Moreover, sleep satisfaction was measured using only one item of the ISI despite the fact that this item was derived from the Swedish version of the ISI, which has good internal consistency (Dragioti, Wiklund, Alföldi, & Gerdle, 2015). Both groups had the significantly highest barriers to pain management at BL. Self‐gathering of knowledge (Wong, 2012) and the weekly follow‐ups might have been sources of appropriate information and subsequently reduced barriers in both groups. Our results are in line with a review that concluded that the influence of education on pain management barriers is limited (Oldenmenger et al., 2009).

One explanation for the mainly similar outcomes of the groups might be the amount of attention given. That is, SMS, the weekly follow‐ups and the survey questionnaire, which included both groups, might have influenced the relative impact of our interventions.

During the study period, both groups with ongoing cancer treatment in our study necessarily met regularly with other healthcare professionals – such as oncologists, radiotherapists and dentists – who provided advice according to their treatment as usual. This essential advice together with the interventions of our project might have been experienced as excessive information to make efficient use for the patients and thus might have contributed to a maybe relatively limited impact of our education and SC interventions. In line with a study of Astrup, Rustøen, Miaskowski, Paul, and Bjordal (2015) but in contrast to two other studies (Elting, Cooksley, Chambers, & Garden, 2007; Epstein, Wilkie, Fischer, Kim, & Villines, 2009), the patients in our study reported relatively low pain intensity. This might have constituted a floor effect and thus limited effects of our interventions.

Eight critical core elements of SC education inventions for patients with cancer have been identified (Howell et al., 2017). Several of the core elements were closely observed in our study but some were probably not emphasized enough. Many factors serve as barriers and facilitators to SC (Riegel, Jaarsma, & Strömberg, 2012), and our consideration of these factors may have been insufficient.

We found a tendency, although not significant, to less pain intensity in the intervention group when speaking, eating and talking and when not performing the above‐mentioned activities at approximately the middle of the study period (Figure 2).

The main limitations include the simple method of randomization, which may lead to poor allocation concealment and the lack of blindness, which further accounts for a significant risk of confounding. However, due to the nature of the disease, the blindness in this study was not possible. The relatively low BL pain intensity may clarify why no significant differences were found (i.e. small possibility of improvement) and possibly constitutes an unintended bias related to denial to participate in the study by patients most affected by the HNC. One may argue that nurses are not trained in teaching patients, although in our study the intervention was structured, the TRNs were clinically experienced in the field of pain management for HNC patients and we can assume that our nursing staff was highly qualified. The interventions were manualized but treatment fidelity was not assessed, which might have influenced the accuracy of the delivered interventions (Dragioti, Dimoliatis, Fountoulakis, & Evangelou, 2015) beyond their “home‐made” nature. Hence, threats to internal validity may be present.

The strengths of this study include the very low dropout rates, little missing data and the participants' recruitment from the ordinary flow of patients at a department specialized in pain management at a university hospital. The representativeness of socio‐demographics in our sample was in line with the general socio‐demographic profile of patients with HNC. Thus, we can infer that our results have population validity. However, ecological validity and further generalizability also may be limited due to the relatively less impaired population.

## 5 CONCLUSIONS

The study concluded that all included patients felt relatively healthy during and after RT. The patients generally reported low pain and good QoL, mood and sleep. However, it was not possible to confirm that patient education and SC reduced pain intensity or improved QoL, mood and sleep during and after RT treatment for HNC.

## 6 CLINICAL IMPLICATIONS AND FUTURE RESEARCH

The study's methodology is based on the structure and the continuity of the personal meeting between the patient and the caregiver and close reporting of symptoms (i.e. pain, QoL, mood and sleep). This applies to both the control and intervention groups (i.e. irrespective of patient education). A secondary effect of the study method probably encouraged the patient to pay attention to perceived symptoms and thus give the caregiver the opportunity to initiate adequate pain management in time. The study shows that it is not primarily pain education that the patient needs. Future research should include the identification of other needs that arise during the cancer treatment and how to optimize treatment for the patient's pain management and QoL.

## AUTHOR CONTRIBUTIONS

ASS and BL: Study conception and study design. ED, BL ASS and GL: Data analyses and manuscript drafting. All authors discussed the results, commented on the manuscript in different versions and approved the current version of the manuscript.

## RESEARCH ETHICS COMMITTEE APPROVAL

All procedures performed in studies involving human participants were in accordance with the ethical standards of the institutional and/or national research committee. (Medical Ethical Board of Linköping University diary number 2014/356‐31) and with the 1964 Helsinki declaration and its later amendments or comparable ethical standards. Informed consent was obtained from all participants included in the study.
