# Supplementary material for: Are patient education and self‐care advantageous for patients with head and neck cancer? A feasibility study
Source: Nurs Open. 2019 Aug 24;6(4):1528–41. doi: 10.1002/nop2.361 (PMC6805323; doi:10.1002/nop2.361)
Supplement: Supplementary file 1 [file NOP2-6-1528-s001.docx]

### Appendix 1

Policies of pharmacological treatment
Patients with mild (NRS 0-4) and moderate pain (NRS 5-6) were prescribed nonsteroidal anti-inflammatory drug treatment in combination with acetaminophen and with the intention to inhibit the production of substances resulting damage and inflammation

If the pain intensity was moderate or severe (NRS 7-10) and assessed as nociceptive and derived from the oral cavity or pharynx, for example, wounds and/or blisters, a strong long-acting opioid was administered directly.

If breakthrough pain occurred, short-acting morphine was prescribed.

In case of poor response after optimization with opioids and if the pain was evaluated as neuropathic, pregabalin was prescribed. Local anesthetics such as oral lidocaine solution was prescribed against pain in the oral mucosa.
