# Supplementary material for: Are patient education and self‐care advantageous for patients with head and neck cancer? A feasibility study
Source: Nurs Open. 2019 Aug 24;6(4):1528–41. doi: 10.1002/nop2.361 (PMC6805323; doi:10.1002/nop2.361)
Supplement: Supplementary file 2 [file NOP2-6-1528-s002.docx]

### Appendix 2

Written information on Self-care for the intervention group.

**To maintain flexibility and well-being, you can do the following (1–8)**

**1. Walks or other activity**

It is good to continue with physical activity that you previously used to do. If this is not possible, take daily walks at a comfortable pace, about 20 minutes a day.

**2. Flexibility and head, throat and shoulders**

To maintain head, throat and neck flexibility, you can do the three following exercises 5–10 times a day.

- Rotate your arms in a large circle forward-upward-backward.
- Put your hands on your thighs, inhale and raise your chest toward the ceiling. Breathe out and roll your shoulders forward without leaning forward.
- Pull in your chin so that your neck is extended, nod your head a few times. Then turn the head to the side, roll your head down over your chest; lift up on the other side, repeat. Keep your chin pulled in.

**3. Open mouth wide to prevent stiffness in your jaw**

To prevent tightness and stiffness in your jaw, it is good to open your mouth wide for a maximum of 20 seconds. Do this ten times in the morning and ten times in the evening.

**4. Swallowing and tongue exercises**

If you have a probe inserted to your stomach, it is important that you try to maintain your ability to swallow. Do this by drinking small sips of water often.

**5. Rest during the day**

It is common to be more tired when receiving radiation therapy. You may need to lay down to rest or sleep once or more a day. Try to limit your rest to 20 min each time to avoid disturbing your night sleeping pattern.

**6. Raise the head of your bed**

Raising the head of your bed or using several pillows is recommended as being horizontal often increases pain since radiated tissue can swell.

**7. In case of sleep problems**

In the evening it is good to lay down for the night when you are tired and do not try to stay awake. Try to get up about the same time each day, even if it took time to fall asleep the night before. If you are having trouble falling asleep in the evening and feel tired during the day, rest in bed (as above) but try to avoid falling asleep. Minimise drinking liquids before bedtime; avoid caffeine and alcohol. If you do not fall asleep after 20 minutes, it is better to get up again.

**8. In case of anxiety**

If you feel anxious, you may want to speak to someone you feel comfortable confiding in. This could be a family member, a friend or someone else. Writing down what you experience and feel can help you determine what is causing the anxiety. You are welcome to contact us at the Pain Relief Clinic. We are here for you and can also help arrange contact with another person to speak to when needed.

**In addition to your prescriptions and advice about pain medications you receive due to mouth and throat pain, try doing some of the following (9–11)**

**9. Dry Mouth**

It is important to keep your mouth moist and have water close at hand.

If you sleep with your mouth open and wake up several times a night because of dryness and secretions, have water by your bed so you can easily moisten your mouth and clean away the secretions.

If needed, the saliva substitute recommended by your dentist may be used for dry mouth.

*Recipe for saliva substitute:*

1 litre of water

1 teaspoon of table salt

1 teaspoon of vegetable oil (rapeseed oil or corn oil)

Boil the water with the salt. Can be stored in the refrigerator up to a week. Mix 1 dl salt solution and 1 teaspoon of oil. Beat with a fork to mix the oil with the salt solution. Rinse your mouth for at least one minute and spit out the solution. This can be repeated every half hour if necessary.

**10. Mouthwash with honey**

Dissolve a teaspoon of honey (not liquid honey) in a cup of lukewarm water. Rinse your mouth for at least one minute and spit out the solution. Alternatively, let a teaspoon of honey melt in your mouth. At most 3–4 times a day

**11. Mouthwash with cream or oil**

Rinse your mouth for one minute with a tablespoon of cream or vegetable oil.

**The following relieve mouth and throat pain (12–14)**

**12. Non-prescription mouth rinse**

Aftex Aloclair® mouth rinse (contains aloe vera) is available at the chemist. Rinse your mouth for at least one minute and spit out the solution. Use 3–4 times a day, or as often as needed.

**13. Cooling**

Sucking on crushed ice or ice cream relieves mouth soreness and pain. Can be done as often as needed.

**14. Mouthwash with morphine**

This treatment is done the first time in consultation with the Pain Relief Clinic. A morphine tablet, 20 mg, is dissolved in 1 teaspoon of water + 1 tablespoon cream. Rinse your mouth for at least one minute and spit out the solution.
