# Supplementary material for: Are patient education and self‐care advantageous for patients with head and neck cancer? A feasibility study
Source: Nurs Open. 2019 Aug 24;6(4):1528–41. doi: 10.1002/nop2.361 (PMC6805323; doi:10.1002/nop2.361)
Supplement: Supplementary file 3 [file NOP2-6-1528-s003.docx]

### Appendix 3

Translation procedure of the questionnaire BQ-II

The BQ-II was translated into Swedish using an iterative forward-backward process. Two public translators – both authorized by the Swedish Legal, Financial, and Administrative Services Agency – translated the BQ-II: a native Swedish speaker translated the English to Swedish and a native English speaker translated the Swedish back into English without access to the original. The two translators did not communicate with each other during the process.

After reviewing the English to Swedish translation, two members of the project decided the participants would have no trouble understanding the Swedish, so no further revisions were necessary.

The Swedish version of the BQ-II has not yet been checked for either reliability or validity. Since reliability, content, structural validity, and hypothesis testing regarding the BQ-II were quite consistently good across three investigated languages and cultures (Norway, Germany, and Denmark), there is good reason to believe the same is true for the Swedish version.
